# Supplementary material for: Porphyromonas gingivalis Bundled Fimbriae Interact with Outer Membrane Vesicles, Commensals and Fibroblasts
Source: Int J Mol Sci. 2025 Dec 30;27(1):383. doi: 10.3390/ijms27010383 (PMC12785474; doi:10.3390/ijms27010383)
Supplement: Supplementary file 1 [file ijms-27-00383-s001.zip › Supplementary Figure S1.docx]

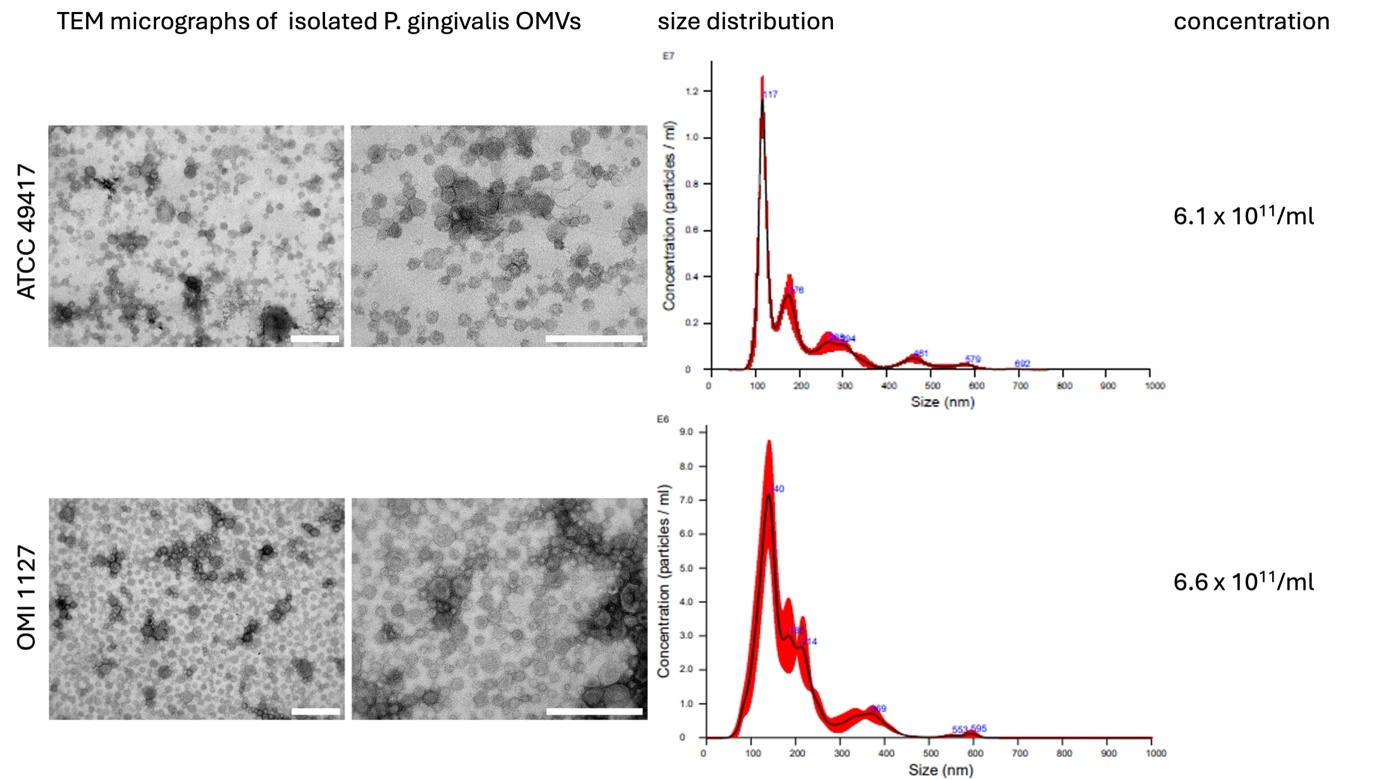


**Figure S1.** Analysis data of OMV preparations from strain ATCC 49417 (OMI 1071) and OMI1127. Size distribution and concentration were determined by NTA. Scalebar corresponds to 500nm.
